# P-825. Short Versus Long Treatment Duration for Streptococcal Bloodstream Infections

**DOI:** 10.1093/ofid/ofae631.1017

**Published:** 2025-01-29

**Authors:** Emily S Spivak, Julie Gray, Chong Zhang, Ali Earl

**Affiliations:** University of Utah School of Medicine, Salt Lake City, Utah; Yale New Haven Hospital, New Haven, Connecticut; University of Utah, Salt lake city, Utah; University of Utah, Salt lake city, Utah

## Abstract

**Background:**

The rise in antibiotic resistance, antimicrobial-related harms and emerging data have prompted a shift towards shorter antibiotic durations. However, there is a lack of data to inform durations of therapy for Streptococcal bloodstream infections (BSI). The need for clearer guidance led us to compare outcomes between short and long antibiotic durations for uncomplicated Streptococcal BSI with the goal of informing local and national practice.

**Figure:**
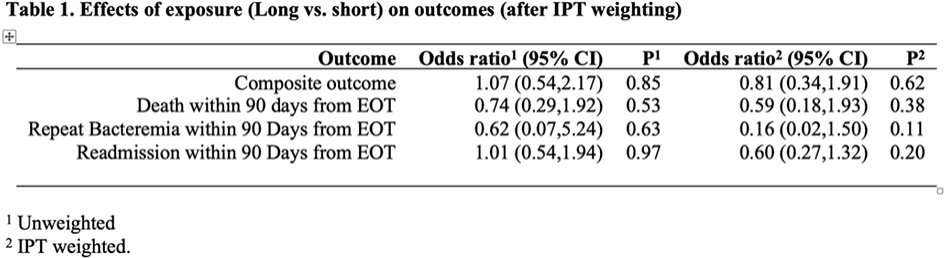

Effects of Long vs. Short Antibiotic Durations

**Methods:**

This was a retrospective cohort study of unique adult patients admitted to University of Utah from January 1, 2014 to July 15, 2023 with uncomplicated Streptococcal BSI, defined as negative follow-up blood cultures, clinical resolution by day 3, absence of metastatic foci, source control if indicated, and no other complications. We used inverse probability of treatment weighting to estimate the average treatment effects (ATE) of in vitro active antibiotics administered for > 10 days (long duration) versus < 10 days (short duration), adjusting for pretreatment variables. The primary outcome was a composite of recurrent bacteremia, all-cause mortality, and readmissions at 30 days from end of treatment (EOT). Secondary outcomes were 90-day all-cause mortality and recurrent bacteremia from EOT.

**Results:**

209 patients met criteria for inclusion. The median duration in the long and short groups were 15 (IQR, 14-17) and 8 days (IQR, 7-9.75), respectively. In the propensity score weighted analysis (Table 1), the ATE of long versus short duration was not significant on the composite primary outcome (21% vs 22%; OR= 0.81 [95% CI: 0.34 to 1.91], p=0.62) or any secondary outcomes.

**Conclusion:**

We found no appreciable difference in outcomes between patients treated with >10 days of antibiotics compared to those treated with <10 days for uncomplicated Streptococcal BSI. Given low absolute rates of mortality and recurrent bacteremia, paired with no appreciable difference related to treatment duration, it is reasonable to recommend shorter durations. Given limitations of this retrospective analysis, future research is needed to confirm our findings; however, our data could inform antimicrobial stewardship interventions aimed at decreasing treatment durations for uncomplicated Streptococcal BSI.

**Disclosures:**

**All Authors**: No reported disclosures

